# The pharmacokinetics of dexmedetomidine in patients with obstructive jaundice: A clinical trial

**DOI:** 10.1371/journal.pone.0207427

**Published:** 2018-11-14

**Authors:** Jin-Chao Song, Hao Gao, Hai-Bo Qiu, Qian-Bo Chen, Mei-Hua Cai, Ma-Zhong Zhang, Zhi-Jie Lu

**Affiliations:** 1 Department of Anesthesiology, Eastern Hepatobiliary Surgery Hospital, Second Military Medical University, Shanghai, China; 2 Department of Anesthesiology, Shanghai Shuguang Hospital, University of Traditional Chinese Medicine, Shanghai, China; 3 Department of Anesthesiology, Shanghai Children’s Medical Center, Shanghai Jiao Tong University School of Medicine, Shanghai, China; Cleveland Clinic, UNITED STATES

## Abstract

**Objectives:**

Dexmedetomidine, a highly selective central α_2_-agonist, undergoes mainly biotransformation in the liver. The pharmacokinetics of dexmedetomidine were significantly affected by hepatic insufficiency. The clearance of dexmedetomidine in patients with severe hepatic failure decreased by 50% compared with controls. We tested the hypothesis that the pharmacokinetics of dexmedetomidine would be affected by obstructive jaundice. The prospective registration number of clinical trial is ChiCTR-IPR-15007572.

**Methods:**

18 patients with obstructive jaundice and 12 non-jaundiced patient controls received dexmedetomidine, 1 μg/kg, over 10 min. Arterial blood samples were drawn before, during, and up to 5 h after the infusion. Plasma dexmedetomidine concentrations were determined by 1290 infinity high performance liquid chromatography coupled with 6470 tandem mass spectrometry. The relevant pharmacokinetic parameters were calculated by non-compartmental analysis using Phoenix WinNonlin 7.0.

**Results:**

Plasma clearance of dexmedetomidine was decreased by 33.3% in the obstructive jaundice group as compared with the control group (0.0068±0.0017 vs. 0.0102±0.0033 L/kg/min; *P* = 0.002). Volume of distribution was decreased by 29.2% in the obstructive jaundice group as compared with the control group (1.43±0.58 vs. 2.02±0.84 L/kg; *P* = 0.041).

**Conclusions:**

This study demonstrates that the clearance and distribution volume of dexmedetomidine were decreased in patients with obstructive jaundice. It may be advisable to adjust the dose of dexmedetomidine in those patients.

## Introduction

Dexmedetomidine is a highly selective central α_2_-agonist with analgesic and sedative properties. In addition, dexmedetomidine decreases anesthetic requirements [1–3]. Dexmedetomidine undergoes mainly biotransformation in the liver with limited unchanged dexmedetomidine excreted in urine or feces [4–6]. Some degree of hepatic dysfunction (e.g., changed drug-metabolizing enzyme systems, impaired biliary excretion, hypoalbuminemia), caused by obstructive jaundice, in general is to be expected. However, pharmacokinetics of dexmedetomidine in patients with obstructive jaundice have not been determined. This study was designed to compare the dexmedetomidine pharmacokinetics in patients with obstructive jaundice with those in patients without obstructive jaundice. We hypothesized that the pharmacokinetics of dexmedetomidine would be affected by obstructive jaundice.

## Methods

This study was approved by the Committee on Ethics of Biomedicine Research, Eastern Hepatobiliary Surgery Hospital, Changhai Rd., No. 225, Shanghai, China (EHBHKY-2014-03-002) prior to its start. The registration number of clinical trial is ChiCTR-IPR-15007572. A total of 30 ASA I/II/III patients undergoing scheduled bile duct surgery, aged 40–70 years and weighing 45–80 kg, were recruited in this study during 2015. Written consent was obtained from all subjects. Patients with known or suspected cardiac, pulmonary, renal, or metabolic disease, weight greater than +/-30% of ideal, and patients on any form of analgesic or neuro-modulating medication were excluded from this study. Patients were divided into two groups according to total serum bilirubin (TBL): an obstructive jaundice group (TBL > 17.1μmol/L, n = 18); a control group (TBL < 17.1 μmol/L, n = 12).

After overnight fasting, a cannula (CV-501-20, Central Venous Catheter, Ltd, Singapore) was inserted into an internal jugular vein for drug administration and fluid replacement. A radial artery catheter (20G, BL BRUN B.) was also inserted under local anesthesia for measuring blood pressure and blood sampling. All patients had the following monitors applied: blood pressure, heart rate, ECG, oxyhemoglobin saturation, and end-tidal carbon dioxide continuously throughout the study (Philips HP Viridia 24/26 M1205A). Lactated Ringer’s solution of 250 mL was infused before anaesthesia if central venous pressure (CVP) was less than 5 mmHg. An IV infusion of lactated Ringer’s solution (1 mL/kg/h) was maintained. Dexmedetomidine 1 μg/kg was infused over 10 min with a Graseby 3500 syringe pump. Arterial blood samples (3 ml) were drawn: before the dexmedetomidine administration; at 0.5, 1, 2, 3, 5 and 10 min during the dexmedetomidine administration; and at 2, 5, 10, 20, 30, 50, 80, 110, 170, 230 and 290 min after the infusion stopped. At 10 min after the infusion stopped, anaesthesia was induced with propofol (1.5–2.0 mg/kg), sufentanil (20 μg) and muscle relaxation rocuronium (N.V.Organon) (50–75 mg). After tracheal intubation, anaesthesia was maintained with 2–4% sevoflurane in oxygen and by bolus administration of sufentanil (10 μg) and rocuronium (25–50 mg) as required throughout the procedure. The depth of anesthesia was monitored using BIS and controlled at 40–50 by fine-adjusting anesthetic agents during operation. Intra-operative intravascular volume management was targeted according to CVP (5–12 mmHg). Blood products (fresh frozen plasma and packed red blood cells) were transfused whenever it was necessary to maintain a haemoglobin value > 9.0 g/dL. The room temperature was controlled at 22–24°C.

Blood samples (using lithium heparin to anticoagulate) were kept on ice until centrifugation within 30 minutes, and plasma samples were stored at -80°C. Plasma dexmedetomidine concentrations were determined by 1290 infinity high performance liquid chromatography coupled with 6470 tandem mass spectrometry (iHPLC-MS/MS, Agilent Technologies, Santa Clara, CA, USA) with a lower limit of quantitation of 10 pg/mL. The intraday and interday coefficients of variation were 0.7% to 5.9% and 2.2% to 5.8%, respectively, for dexmedetomidine concentrations in the range of 0.01 to 10 ng/mL. The main pharmacokinetic parameters: clearance (CL), terminal elimination half-life (T_1/2_), Cmax, the area under the concentration-time curve (AUC) from zero to the last measurable plasma concentration point (AUC_0-t_), AUC from 0 to infinity (AUC_0-∞_), mean residence time (MRT), and volumes of distribution (V_d_) were calculated by non-compartmental analysis using Phoenix WinNonlin 7.0 (Pharsight Corporation, Mountain View, CA, USA). The operator of drug concentrations analysis were blinded to the grouping.

Group sample size was calculated based on the result of a pilot study, in which the mean CL was 0.007±0.002 (n = 5) L/kg/min in the obstructive jaundice group and 0.010±0.002 (n = 5) L/kg/min in the control group. 8 samples for each group met the requirement of power = 0.8 and α = 0.05 (two-sided) [7].

Measurement data are expressed as arithmetic means ± standard deviation/standard error (normal distribution data) or median and interquartile range (non-normal distribution data). Statistical analysis was analyzed with independent 2-sample t-test (2-sided) or non-parametric test. The count data were compared using chi-square. Linear mixed model was used to analysis the repeated measurements with missing values in our study. Correlation analysis has been used to fit a linear model. P<0.05 was considered statistically significant. All analyses were conducted using SPSS 17.0 (SPSS Inc., Chicago, IL). Figures were made using Graph Pad Prism 5.

## Results

The demographic and clinical characteristics of the two groups are presented in Table 1. There were no significant differences in gender, age, body height, body weight, albumin, serum creatinine (SCR), blood urea nitrogen (BUN), prothrombin time (PT) and international normalized ratio (INR) (*P*>0.05). The values of TBL, alanine aminotransferase (ALT) and aspartate aminotransferase (AST) in the obstructive jaundice group were higher than those in the control group (*P*<0.05).

**Table 1:** Patient characteristics and preoperative laboratory values.

	Obstructive jaundice group (n = 16)	Control group (n = 11)	*P* value
Gender male/female	8/8	4/7	0.696
Age (years)	57.5±7.5	61.5±5.5	0.149
Body height (cm)	160.4±6.5	160.5±6.1	0.995
Weight (kg)	63.0 (54.8, 65.8)	56.0 (51.5,74.0)	0.610
Bilirubin (μmol/L)	119.9±59.0	11.9±4.5	**<0.001**
ALT (U/L)	115.0 (59.8, 205.0)	26.0(17.0, 62.0)	**<0.001**
AST (U/L)	85.5 (35.5, 150.5)	23.0 (19.0, 50.0)	**0.001**
Albumin (g/L)	37.6±2.7	38.4±9.2	0.777
SCR (mmol/L)	62.7±14.5	62.4±15.7	0.956
BUN (mmol/L)	5.2 (3.7, 5.8)	4.19 (3.60, 5.89)	0.680
INR	0.99±0.13	0.92±0.09	0.143
PT (sec)	10.4 (10.0, 10.8)	10.8 (10.0, 10.6)	0.318
Malign disease			
Carcinoma of head of pancreas	3	0	
Gallbladder carcinoma	1	5	
Hilar bile duct cholangiocarcinomas	3	1	
Carcinoma in the middle and distal bile duct	7	4	
Intrahepatic bile duct cholangiocarcinomas	2	1	

Normal distribution data are expressed as means±standard deviation(SD); Non-normal distribution data are expressed as median and interquartile range. *P*<0.05 was considered statistically significant. ALT, alanine aminotransferase; AST, aspartate aminotransferase; BUN, blood urea nitrogen; SCR, serum creatinine; INR, international normalized ratio; PT, prothrombin time.

**Fig 1:**
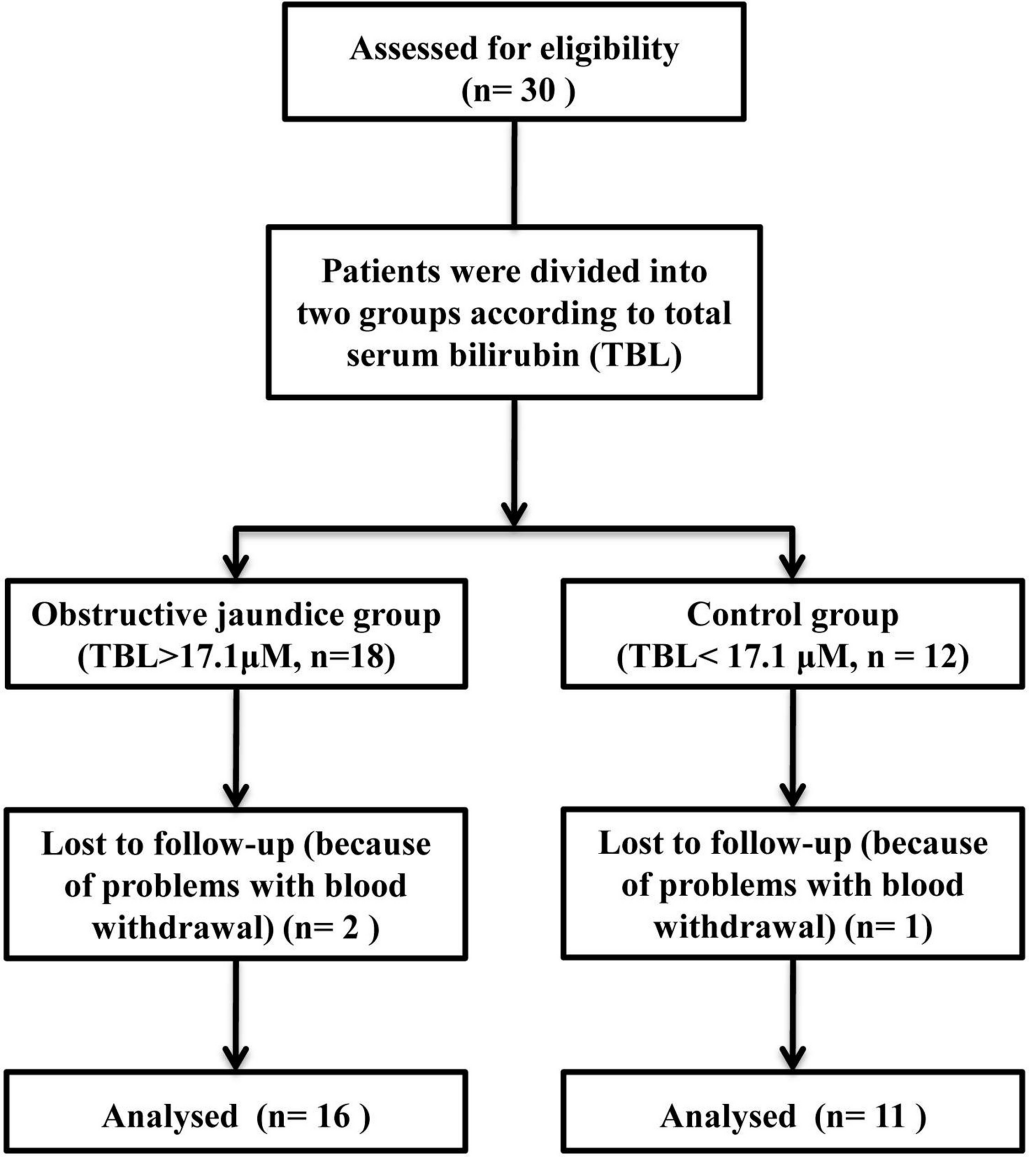
Flow diagram.

**Fig 2:**
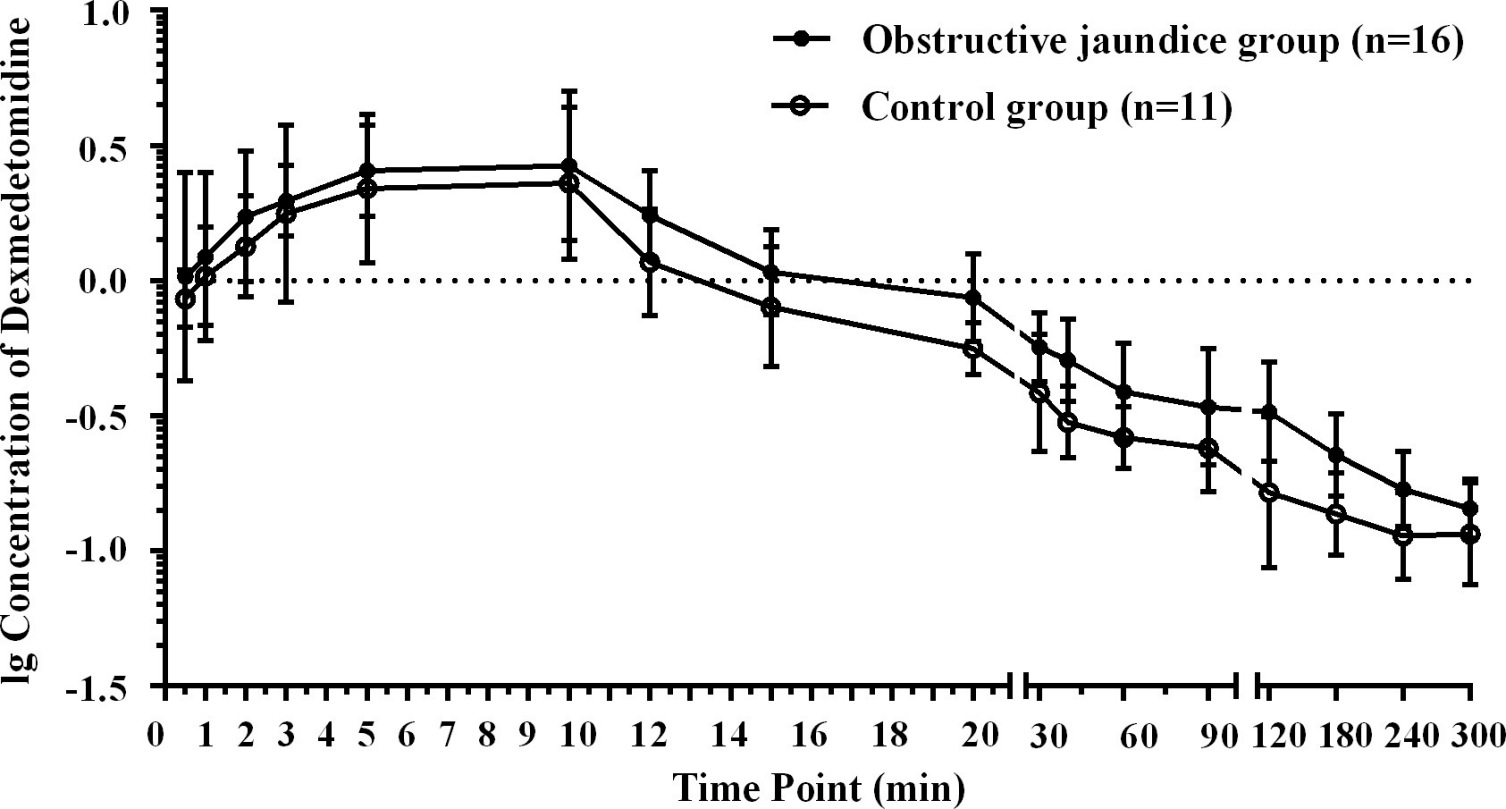
Mean dexmedetomidine plasma concentration on a logarithmic scale versus time data for both groups. Data are expressed as arithmetic means ± standard error (SEM).

**Fig 3:**
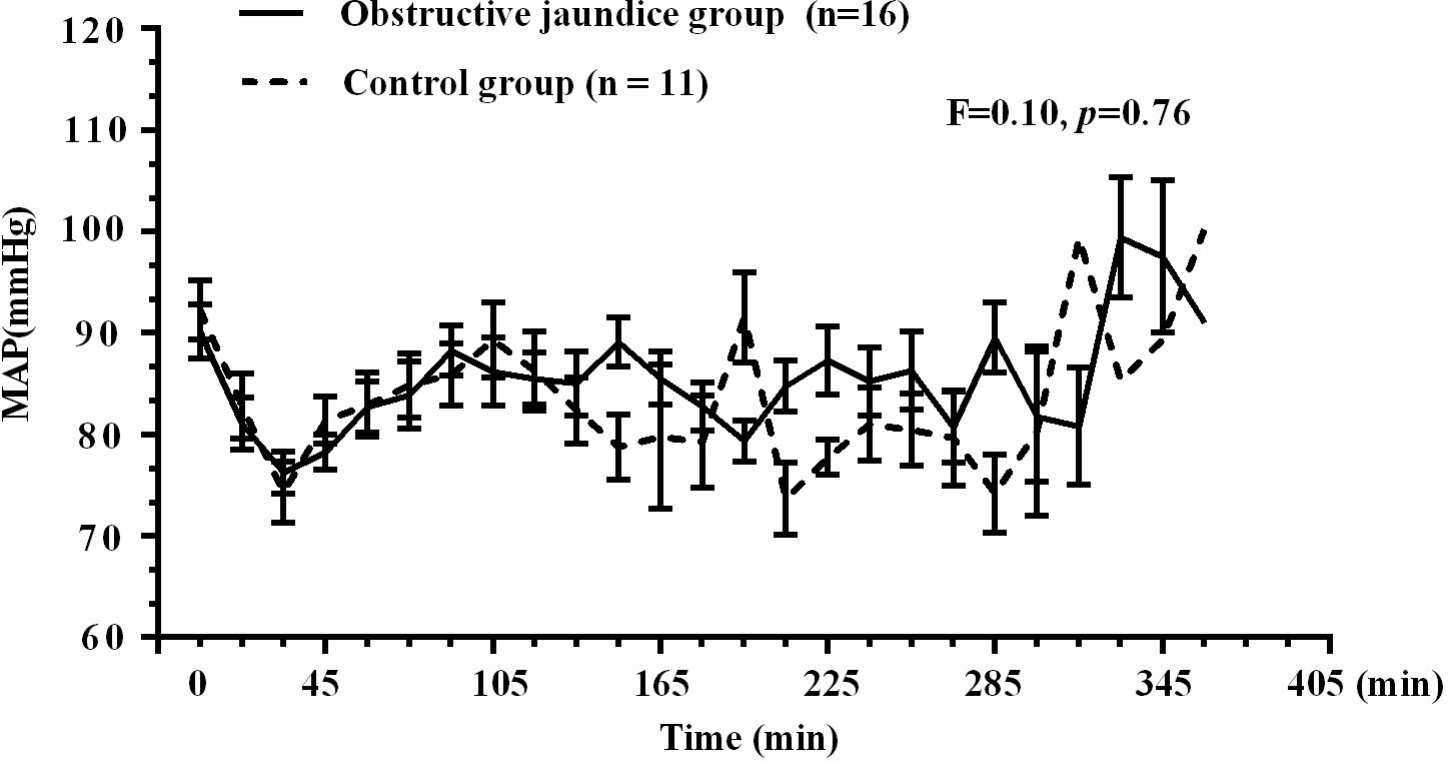
Hemodynamic changes of patients during surgeries for both group (*p* = 0.76).

Anaesthesia and recovery proceeded without complications (allergies, asthma, laryngospasm, etc.) in all patients. The data from 3 patients (two in the obstructive jaundice group and one in the control group) were unsuitable for pharmacokinetic analysis because of problems with blood withdrawal; thus, 16 in the obstructive jaundice group and 11 in the control group were included in the kinetic analysis (Fig 1. Flow diagram). Fig 2 showed the mean dexmedetomidine plasma concentration on a logarithmic scale versus time data for both groups. Linear mixed model in SPSS was used for analysis of blood pressure during surgeries. The hemodynamics (MAP) of two groups were comparable in the perioperative period (F = 0.10, *p* = 0.76). Fig 3 showed the hemodynamic changes of patients during surgeries for both group.

The pharmacokinetic parameters were summarized in Table 2. Plasma CL of dexmedetomidine was decreased by 33.3% in the obstructive jaundice group as compared with the control group (0.0068±0.0017 vs. 0.0102±0.0033 L/kg/min; *P* = 0.002); Volume of distribution was decreased by 29.2% in the obstructive jaundice group as compared with the control group (1.43±0.58 vs. 2.02±0.84 L/kg; *P* = 0.041). In contrast, Cmax, AUC_0-t_ and AUC_0-∞_ in the obstructive jaundice group were increased compared with the control group. There was no significant difference in T_1/2_ and MRT_0-∞_ between obstructive jaundice group and control group (P>0.05).

**Fig 4:**
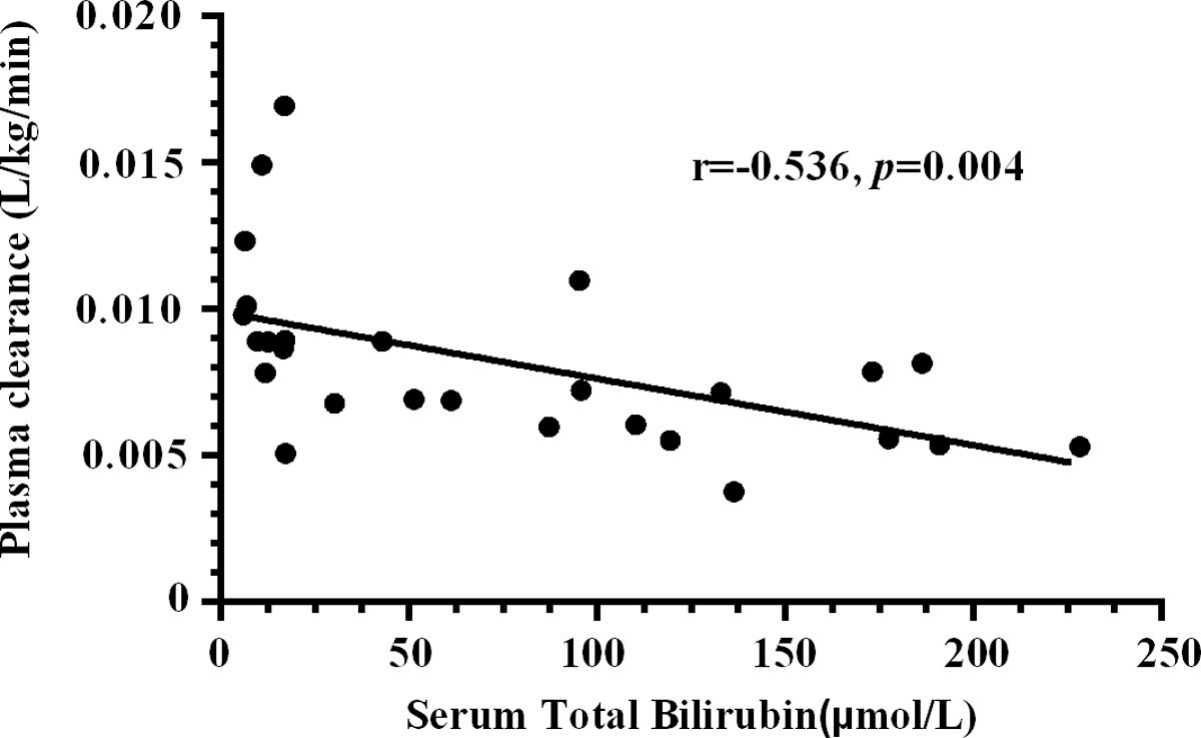
Correlation analysis reveals a significant correlation between serum total bilirubin and clearance of dexmedetomidine (*P* < 0.05).

Correlation analysis showed a significant negative correlation between serum total bilirubin and clearance of dexmedetomidine (r = -0.536, *p* = 0.004) (Fig 4).

**Table 2:** Pharmacokinetic variable of each group.

	Obstructive jaundice group (n = 16)	Control group (n = 11)	*P* value
CL (L/kg/min)	0.0068±0.0017	0.0102±0.0033	**0.002**
T_1/2_ (min)	141 (106, 195)	138 (102, 155)	0.790
Cmax (ng/ml)	3.29 (2.68, 4.28)	2.60 (1.96, 2.77)	**0.020**
AUC_0-t_ (ng/ml/min)	112.16 (96.76, 132.32)	81.16 (58.21, 91.19)	**0.004**
AUC_0-∞_ (ng/ml/min)	156.05±40.06	107.58±36.38	**0.004**
MRT_0-∞_ (min)	164.58(114.25, 245.81)	143.06 (104.75, 178.44)	0.442
Vd (L/kg)	1.43±0.58	2.02±0.84	**0.041**

Normal distribution data are expressed as mean±standard deviation(SD); Non-normal distribution data are expressed as Median and interquartile range. P<0.05 was considered statistically significant. CL, clearance; T_1/2_, terminal elimination half-life; AUC_0-t_, the area under the concentration-time curve from zero to the last measurable plasma concentration point; AUC_0-∞_, AUC from 0 to infinity; MRT, mean residence time and Vd, volumes of distribution.

## Discussion

We have studied the effect of obstructive jaundice on dexmedetomidine pharmacokinetics and found out that plasma CL of dexmedetomidine in patients with obstructive jaundice is decreased significantly compared with those in non-jaundiced controls. In addition, there was a significant negative correlation between TBL and CL.

Dexmedetomidine has a rather high extraction ratio and it is mainly biotransformed in the liver, with limited renal elimination clearance [5,6], its CL is dependent on total blood supply to the liver. Obstructive jaundice may affect (reduce) the liver blood flow through various mechanisms [8–10]. In a dog model, hepatic arterial flow and portal venous flow after biliary obstruction and subsequent drainage were measured using implantable transit time ultrasonic flow-meters. It was found that biliary obstruction could result in significant changes in liver circulation, and biliary drainage could facilitate recovery from these changes [8]. Gram-negative sepsis is a serious complication in patients with obstructive jaundice. In a rat model, it was demonstrated that chronic biliary obstruction increased the hepatic microvascular response to low doses of endotoxin [9], and that exaggerated hepatic microcirculatory dysfunction during sepsis may contribute to the development of liver injury. It was reported that the hepatic sinusoidal microcirculation was impaired during endotoxemia [10].

Dexmedetomidine underwent almost complete biotransformation, which involved both direct glucuronidation and cytochrome P450-mediated metabolism. The major metabolic pathways of dexmedetomidine are (1) aliphatic hydroxylation mediated primarily by CYP2A6, (2) direct N-glucuronidation to inactive metabolites, and (3) N-methylation of dexmedetomidine [4]. The pharmacokinetics of dexmedetomidine were significantly affected by hepatic insufficiency. The CL of dexmedetomidine in patients with severe hepatic failure decreased by 50% compared with controls [11]. Obstructive jaundice may result in hepatic cell damage and hepatosis through various mechanisms [12,13]. The drug-metabolizing enzyme systems may impaired in patients with obstructive jaundice. Furthermore, dexmedetomidine inhibited cytochrome P450 enzyme systems (CYP2D6) in human liver microsomes in vitro [14]. Therefore, we thought that the change of liver blood flow caused by obstructive jaundice and the impaired drug-metabolizing enzyme systems caused by hepatic dysfunction contribute to the decrease of plasma CL of dexmedetomidine.

We previously reported that the pharmacokinetics of propofol are not affected markedly by obstructive jaundice [15]. The difference between dexmedetomidine and propofol pharmacokinetics in patients with obstructive jaundice may be attributed in part to the different metabolic pathways. Dexmedetomidine is mainly biotransformed in the liver. However, propofol can be biotransformed not only in the liver but also in other organs, such as kidney and intestine. It is possible that extrahepatic metabolism of propofol play a more important role in the patients with obstructive jaundice than without obstructive jaundice.

Our study has 2 limitations. Firstly, there is small sample size in our study, which may have limited the precision of our pharmacokinetics analyses. Secondly, after dexmedetomidine administration, anaesthesia was maintained with 2–4% sevoflurane; therefore, it is difficult to observe the differences in dexmedetomidine pharmacodynamics (analgesic and sedative properties, and the effect on hemodynamics) between patients with and without jaundice.

In conclusion, our study demonstrates that the pharmacokinetics of dexmedetomidine was affected by obstructive jaundice. The CL of dexmedetomidine in patients with obstructive jaundice decreased compared with controls. These data suggest that it may be advisable to adjust the dose of dexmedetomidine in patients with obstructive jaundice.

## Supporting information

S1 File “CONSORT_CHECKLIST”. (DOC)

S2 File “Data”. (XLSX)

S3 File “The trial study protocol”. (DOCX)

S4 File “The trial study protocol(Chinese)”. (PDF)
