# Supplementary material for: The pharmacokinetics of dexmedetomidine in patients with obstructive jaundice: A clinical trial
Source: PLoS One. 2018 Nov 14;13(11):e0207427. doi: 10.1371/journal.pone.0207427 (PMC6235379; doi:10.1371/journal.pone.0207427)
Supplement: S3 File — (DOCX) [file pone.0207427.s003.docx]

**The Trial Study Protocol**

**1. Purpose：**

We tested the hypothesis that the pharmacokinetics of dexmedetomidine would be affected by obstructive jaundice.

**2. Background：**

Dexmedetomidine，a highly selective central α2-agonist with analgesic and sedative properties，undergoes mainly biotransformation in the liver. The pharmacokinetics of dexmedetomidine were significantly affected by hepatic insufficiency. The clearance of dexmedetomidine in patients with severe hepatic failure decreased by 50％ compared with controls.

**3. Patients** **recruitment and grouping：**

A total of 30 ASA I/II/III patients undergoing scheduled bile duct surgery, aged 40-70 years and weighing 45-80 kg, were recruited in this study. Written consent was obtained from all subjects. Patients with known or suspected cardiac, pulmonary, renal, or metabolic disease, weight greater than +/-30% of ideal, and patients on any form of analgesic or neuro-modulating medication were excluded from this study. Patients were divided into two groups according to total serum bilirubin (TBL): an obstructive jaundice group (TBL > 17.1μmol/L, n=18); a control group (TBL < 17.1 μmol/L, n = 12).

4. **Dexmedetomidine administration and Blood samples withdrawal**

Dexmedetomidine 1 µg/kg was infused over 10 min with a Graseby 3500 syringe pump. Arterial blood samples (3 ml) were drawn: before the dexmedetomidine administration; at 0.5，1，2，3，5 and 10 min during the dexmedetomidine administration; and at 2，5，10，20，30，50，80，110，170，230 and 290 min after the infusion stopped. At 10 min after the infusion stopped, anaesthesia was induced with propofol (1.5-2.0 mg/kg), sulfentanyl (20 μg) and muscle relaxation rocuronium (50-75 mg). After tracheal intubation, anaesthesia was maintained with 2-4% sevoflurane in oxygen and by bolus administration of sulfentanyl (10 μg) and rocuronium (25-50 mg) as required throughout the procedure. The depth of anesthesia was monitored using BIS and controlled at 40-50 by fine-adjusting anesthetic agents during operation. Intra-operative intravascular volume management was targeted according to CVP (5-12 mmHg). Blood products (fresh frozen plasma and packed red blood cells) were transfused whenever it was necessary to maintain a haemoglobin value > 9.0 mg/dl. The room temperature was controlled at 22-24°C.

**5**. **Dexmedetomidine concentrations analysis：**

Blood samples (using lithium heparin to anticoagulate) were kept on ice until centrifugation, and plasma samples were stored at -80°C. Plasma dexmedetomidine concentrations were determined by 1290 infinity high performance liquid chromatography coupled with 6470 tandem mass spectrometry (iHPLC-MS/MS, Agilent Technologies, Santa Clara, CA, USA) with a lower limit of quantitation of 10 pg/mL.

**7. Dexmedetomidine pharmacokinetics analysis：**

Dexmedetomidine pharmacokinetics analysis is performed by using the software program NONMEM first. If there is no suitable compartment models for pharmacokinetics of dexmedetomidine, the relevant pharmacokinetic parameters will be calculated by non-compartment model analysis using Phoenix WinNonlin 7.0.

**8. Statistical analysis:**

All data in the tables and text are presented as mean ± SD or number (n). Continuous outcomes with normal distribution were analyzed with independent 2-sample t-test (2-sided). The count data were compared using chi-square. All analyses were conducted using SPSS 17.0 (SPSS Inc., Chicago, IL). Figures were made using GraphPad Prism 5.

**The Trial Study Flow Diagram:**

Patients recruitment

Patients were grouped according to TBL

Control group

Obstructive jaundice group

**Statistical analysis**

Dexmedetomidine 1 µg/kg was infused over 10 min with a Graseby 3500 syringe pump.铵。术中不用右美托咪定。麻醉深度BIS控制在40-50。

Dexmedetomidine 1 µg/kg was infused over 10 min with a Graseby 3500 syringe pump.铵。术中不用右美托咪定。麻醉深度BIS控制在40-50。

Arterial blood samples (3 ml) were drawn: before the dexmedetomidine administration; at 0.5，1，2，3，5 and 10 min during the dexmedetomidine administration; and at 2，5，10，20，30，50，80，110，170，230 and 290 min after the infusion stopped.

Arterial blood samples (3 ml) were drawn: before the dexmedetomidine administration; at 0.5，1，2，3，5 and 10 min during the dexmedetomidine administration; and at 2，5，10，20，30，50，80，110，170，230 and 290 min after the infusion stopped.

Plasma dexmedetomidine concentrations were determined by HPLC

Dexmedetomidine pharmacokinetics analysis

Dexmedetomidine pharmacokinetics analysis

Plasma dexmedetomidine concentrations were determined by HPLC
