# Supplementary material for: The pharmacokinetics of dexmedetomidine in patients with obstructive jaundice: A clinical trial
Source: PLoS One. 2018 Nov 14;13(11):e0207427. doi: 10.1371/journal.pone.0207427 (PMC6235379; doi:10.1371/journal.pone.0207427)

临床研究方案

研究目的：

阐明梗阻性黄疸患者使用右美托咪定的药效动力学；明确梗阻性黄疸患者使用右美托咪定的药代动力学。从而更加合理地指导临床用药，提高围术期手术与麻醉的安全，最大程度地降低此类手术的并发症及死亡率。

立题依据：

梗阻性黄疸作为肝胆外科常见的一种临床表现，主要是由肝内、外胆管的机械性梗阻造成胆汁郁积而引起的，常见于胰头癌、肝门部胆管癌等疾病。梗阻性黄疸对机体正常生理功能的影响很广泛，血清内胆红素浓度增高（高胆红素血症）除了肝脏功能本身的损害以外，心血管系统以及中枢神经系统的功能都遭受一定程度的损害。此类手术的并发症及死亡率很高，有文献［1］报道胰十二指肠切除术术后并发症发生率为23%～57%，手术死亡率为1.9%～15%。梗阻性黄疸患者易并发低血压，并且手术后易发生肾功能衰竭[2,3]发病率在 8～10％，其中病死率高达 70～80％。因此，研究梗阻性黄疸患者的临床常用全麻药物的作用特点，提高对黄疸患者的临床麻醉质量，对于确保围术期手术与麻醉的安全，减少术后并发症，提高生存率具有积极的意义。

以往的研究认为阻塞性黄疸可能主要从以下几个方面影响机体的心血管功能:①阻塞性黄疸可使外周血管对体内血管活性物质（如去甲肾上腺素等）的反应性下降，心脏后负荷降低[4]；②阻塞性黄疸对心脏具有负性肌力和负性变时作用，导致心功能下降[5,6]；③手术过程当中的出血、低血压和麻醉药物可能促进了术后的低血压和肾功能衰竭的发生[7]；④胆盐的利尿和促尿钠排泄作用

可能促成了机体的低血容量状态[8]；⑤内毒素血症本身及其所诱导的 NO 合成的增加导致外周血管舒张[9]。

我们一项 112 例梗阻性黄疸患者术中血流动力学的临床回顾性分析发现梗阻性黄疸病人诱导前后的 SBP 变化剧烈、术中血压、心率低于非黄疸病人，血压、心率也更易波动。阻黄病人对于麻黄碱、多巴胺及阿托品等血管活性药物不敏感，用药的剂量和频率增加[10]。我们还证实了梗阻性黄疸患者的地氟烷 MAC-awake 显著低于正常患者，即黄疸患者对吸入麻醉药的敏感性增强，而且其 MAC-awake 与血浆总胆红素有显著负性相关关系[11]。我们还证实了梗阻性黄疸患者与非阻黄患者相比达到相同镇静深度的依托咪酯的需要量是下降的[12]。我们在临床及动物实验（大鼠）中分别研究了梗阻性黄疸对动脉压力感受反射敏感性（BRS）的影响，发现梗阻性黄疸削弱了动脉压力感受反射功能（交感反射敏感性和迷走反射敏感性均显著下降），且交感迷走失衡，迷走神经相对占优[13]。交感反射敏感性的降低与交感迷走的失衡密切相关。

右美托咪定是高选择性 a2 肾上腺素能受体激动剂，具有中枢性抗交感作用，能产生近似自然睡眠的镇静作用；同时具有一定的镇痛、利尿和抗焦虑作用，对呼吸无抑制，还具有对心、肾和脑等器官功能产生保护的特性，故而在麻醉围术期以及重症患者的镇静中有独特优势[14]。右美托咪定与其他镇静催眠药的作用机制不同，可产生自然非动眼睡眠，在一定剂量范围内机体的唤醒系统功能仍然存在。接受右美托咪定患者 Ramsay≥3 分或 OAA/S≤4 分受到刺激时可观察到觉醒反应。在 ICU 使用可以给患者带来的益处：能够缓解患者的焦虑和烦躁，使患者能够较舒适、安静地接受呼吸机治疗，能够随时被唤醒，配合相应治疗[15]。由于患者是处在自然睡眠下，有利于患者精力的恢复，并存在免疫应答，减少感

染发生率。但是，右美托咪定最常见不良反应为低血压、心动过缓[16]。迷走张力高、糖尿病、高血压、高龄、肝功能或肾功能有损伤的患者更易发生心动过缓，甚至窦性停搏。

右美托咪定与白蛋白结合力为94%，有95%的药物在肝脏经葡萄糖醛酸化，经 CYP2A6 水解以及甲基化后生成非活性代谢产物[17]。肝功能损害可显著影响右美托咪定的药代动力学特性[14]。在肝功能损害的患者体内，右美托咪定达稳态的分布容积增加至 3.2L/kg，消除半衰期延长至 7h，肝脏清除率相对正常肝功能者下降约 50%。

梗阻性黄疸患者是否对右美托咪定具有更高的敏感性？在梗阻性黄疸患者中右美的副作用（低血压、心动过缓）是否会放大？梗阻性黄疸患者的术后肝功能异常是否影响右美的代谢？

研究现状：这些目前在国内外文献未见报道。

主要参考文献

1. Bouvet M，Gamaga RA，Gilpin EA，et a1．Factors influencing survival after resection for periampullary neoplasms．Am J Surg，2000，180：l3-l7.
2. Williams RD, Elliott DW, Zollinger RM: The effects of hypotension in obstructive jaundice. Arch Surg 1960; 81:334-40.
3. Walker JG: Renal failure in jaundice. Proc R Soc Med 1962;55:570-4.
4. Bomzon A, Rosenberg M, Gail D, et al: Systemic hypotension and decreased pressor response in dogs with chronic bile duct ligation (CBDL). Hepatology 1986; 6:595-600.

5. Joubert P: Cholic acid and the heart: In vitro studies of the effect on heart rate and myocardial contractility in the rat. Clin Exp Pharmacol Physiol 1978; 5:9-16.
6. Joubert P: An in vivo investigation of the negative chronotropic effect of cholic acid in the rat. Clin Exp Pharmacol Physiol 1978; 5:1-8
7. Green J, Better OS: Systemic hytension and renal failure in obstructive jaundice-mechanistic and therapeutic aspects. J Am Soc Nephrol. 1995 ;5:1853-71.
8. Finestone H, Fechner C, Levy M: Effects of bile and bile salt infusions on renal function in dogs. Can J Physiol Pharmacol 1984; 62:762-768 .
9. Claria J, Jimenez W, Ros J, et al: Pathogenesis of arterial hypotension in cirrhotic rats with ascites: Role of endogenous nitric oxide. Hepatology 1992; 15:343-9.
10. 宋金超 俞卫锋 杨立群 梗阻性黄疸患者术中血液动力学变化规律的临床分析. 临床麻醉学杂志 2005,21;07.
11. Song JG, Cao YF, Yang LQ, et al. Awakening concentration of desflurane is decreased in patients with obstructive jaundice. Anesthesiology, 2005, 102: 562-565.
12. JC Song, YM Sun, WF Yu. The etomidate requirement is decreased in patients with obstructive jaundice. Anesthesia and analgesia. 2011;113(5):1028-32.
13. Song JG, Cao YF, Sun YM, et al. Baroreflex sensitivity is impaired in patients with obstructive jaundice. Anesthesiology, 2009,111(3):561-5.
14. BHANA N，GOAK L，MCCLELLANK J．Dexmedetomidine[J]． Drugs，2000，59(2)：263-268.

15. PICHOT C，GHIGNONE M，QUINTIN L．Dexmedetomidine and Clonidine：From Second-to-First Line Se dative Agents in the Critical Care Setting[J]．J Intensive Care Med，2011，27 published on line.

16. RIKER R R，SHEHABI Y，BOKESCH P M．Dexmedetomidine vs midazolam for sedation of critically ill patients：a randomized trial [J]．JAMA，2009，301(5)：489—499.

17. TAIJI K．Dexmedetomidine hydrochloride(precedex)，a new sedative in intensive care，its pharmacological characteristics an d clinical study result[J]. Nihon Yakurigaku Zasshi，2004，124(3)： 171—179.

研究内容：

（1）*梗阻性黄疸对右美托咪定的药效动力学的影响。序贯法测定右美托咪定的EC50*

病人选择及分组　选择 ASAI-III级择期手术病人 60 例，有严重心、肝、肺、肾功能不全的患者排除在本研究之外。手术前禁食 6~8h，所有病人均不使用术前药。试验组：30 例择期梗阻性黄疸病人，壶腹周围恶性肿瘤、肝门部胆管癌(TBL>17.1μmol/L)；对照组：30 例普通胆道手术(TBL<17.1μmol/L)。

病人入室后首先监测无创血压、ECG、BIS 和 SpO_2，局麻下一侧桡动脉插管用于监测动脉压和取样，右侧颈内静脉穿刺置管，麻醉诱导罗库溴铵、丙泊酚、舒芬太尼快诱导插管；麻醉维持丙泊酚、瑞芬太尼，根据需要给予罗库溴铵。术中不用右美托咪定。麻醉深度 BIS 控制在 40-50。术中维持循环、CVP 的稳定，以及血红蛋白在正常范围。

手术结束后除了必要时给予肌松拮抗外其余麻醉效应不予拮抗，自主呼吸稳

定后，带气管插管回 ICU，常规给予监测有创桡动脉持续监测血压、ECG 和 SpO2，右侧颈内静脉持续补液林格氏液 1ml/kg/h。

序贯法测定右美托咪定的 EC50

术后患者回恢复室，患者苏醒后拔除气管导管，并进行数字分级评分法 NRS 疼痛评分和 Ramsay 镇静评分，每 5 分钟评一次。当 NRS 评分大于 3 分时，给予右美托咪定持续输注 10 分钟，给药剂量按公斤体重计算。输注完毕后 15 分钟进行 NRS 疼痛评分评价效果，小于等于 3 分为有效，大于 3 分为无效。采用序贯法根据同组（黄疸组或非黄疸组）上一名患者的疗效调整剂量，测定 EC50。各组第一名患者给予 1ug/kg，随后的每一位患者根据同组的前一位的疗效，进行剂量调整(增加或减少剂量)调整间隔为 0.2 ug/kg，最大剂量 2 ug/kg。除了 NRS 疼痛评分外，还进行 Ramsay 镇静评分，同时观察记录患者心率、血压、氧饱和度、右美托咪定的副作用等。对于具体的某一位患者试验结束后，根据实际需要采用吗啡镇痛，并记录 48 小时内的吗啡实际需要量。

统计学处理　计量资料均用均数±标准差表示($\bar{x} \pm s$)，组间比较采用单因素方差分析，$P<0.05$ 为差异有统计学意义。

（2）*梗阻性黄疸病人术后持续输注右美托咪定的药代动力学*

病人选择及分组　选择 ASAI-III级择期手术病人 30 例，有严重心、肝、肺、肾功能不全的患者排除在本研究之外。手术前禁食 6~8h，所有病人均不使用术前药。试验组：18 例择期梗阻性黄疸病人，壶腹周围恶性肿瘤、肝门部胆管癌(TBL>17.1μmol/L)；对照组：12 例普通胆道手术(TBL<17.1μmol/L)。

监测与麻醉过程 (右美托咪定的输注与取样)

病人入室后首先监测无创血压、ECG、BIS 和 SpO2，局麻下一侧桡动脉插管用于监测动脉压和取样，右侧颈内静脉穿刺置管。做好监护等准备工作后，开始输注右美托咪定，输注剂量为 1ug/kg 于 10 分钟内由 Graseby 3500 泵输注。由桡动脉留置管每次抽取 3ml 血标本，抽血时点：输注前、开始输注后（输注期间）的 0.5，1，2，3，5 和 10 分钟；输注结束后的 2，5，10，20，30，50，80，110，170，230 和 290 分钟。 血标本置于冰上，离心后得血浆放入-80°C 冰箱冷冻保存。输注右美完毕后，麻醉诱导药物：丙泊酚 (1.5-2.0 mg/kg), 舒芬太尼(20μg) 和 罗库溴铵(50-75 mg). 插管后麻醉维持 2-4% 七氟醚，根据需要必要时单次给予舒芬太尼 (10μg) 和罗库溴铵 (25-50 mg)。麻醉深度控制在 BIS 50 左右。术中维持循环、CVP 的稳定，以及血红蛋白在正常范围。

右美托咪定血浆浓度测定：

右美托咪定血浆浓度用高效液相(HPLC)色谱仪分析。此部分工作交给本院药剂科药理实验室完成。

药动学模型 用 NONMEM®软件采用三室线性中央室消除模型(图 2)分析药动学参数。选择系统清除率(CL)、K_{10}、K_{12}、K_{21}、K_{13}、K_{31} 等为需要估计的药动学参数。

用标准二阶段法对每位患者的血浆和时间数据均采用二室和三室模型进行拟合，拟合的程序是“非线性扩展的最小二乘回归程序(MKMODEL)”，即：对

每位患者，均对其中央室容积和房室间转运速率常数 kij 进行估计。回归的目标是目标函数(Objective function)最小，即：

$$O = \sum_{i=0}^{n} \frac{\left(Y_i - \hat{Y_i}\right)^2}{V_i} + Ln(V_i)$$

式中 n 为某患者的样本数，Yi 为第 i 个样本实测浓度，$\hat{Y}$为第 i 个样本的预测浓度，Vi 是第 i 个样本的预测变异性，Vi 可根据下列公式计算：

$$V_i = (\sigma \hat{Y})^2$$

σ 为被估计参数的变异性，根据下式计算：

$$\sum_{i}^{n} \frac{\left(Y_i - \hat{Y}_i\right)}{\left(\sigma \hat{Y}_i\right)^2} = n.$$

回归处理；协变量包括年龄、体重、身高、性别均包括在模型中逐一分析。用最大拟然性目标函数(Maximum likelihood objective function，-2LL)评价两室和三室模型的拟合优度，目标函数差异性用卡方检验，自由度为 df=房室数差，df=1 时 p=0.05 或 0.01 的卡方值分别是 3.84 和 6.63，df=2 时 p 值=0.05 和 0.01 的卡方值则分别是 5.99 和 9.21。为估计模型的准确性，我们计算了每个样本的残差(R)、加权残差(WR)和绝对值加权残差(AWR)：

$$R_{ij} = C_{ij} - CP_{ij} \qquad WR_{ij} = \frac{C_{ij} - CP_{ij}}{CP_{ij}} \qquad AWR_{ij} = \frac{\left|C_{ij} - CP_{ij}\right|}{CP_{ij}}$$

Cij 是第 *i* 个个体第 *j* 个测量浓度，*CPij* 为相应的预测值。*WR* 和 *AWR* 的中位数 *MWR* 和 *MAWR* 作为评价模型拟合优度的标准。

统计学处理 计量资料均用均数±标准差表示($\bar{x} \pm s$)，组间比较采用单因素方差分析，P<0.05 为差异有统计学意义。

拟解决的关键问题：

①梗阻性黄疸对右美托咪定的药效动力学的影响。

②梗阻性黄疸对右美托咪定的药代动力学的影响。

药代动力学部分研究的流程图：

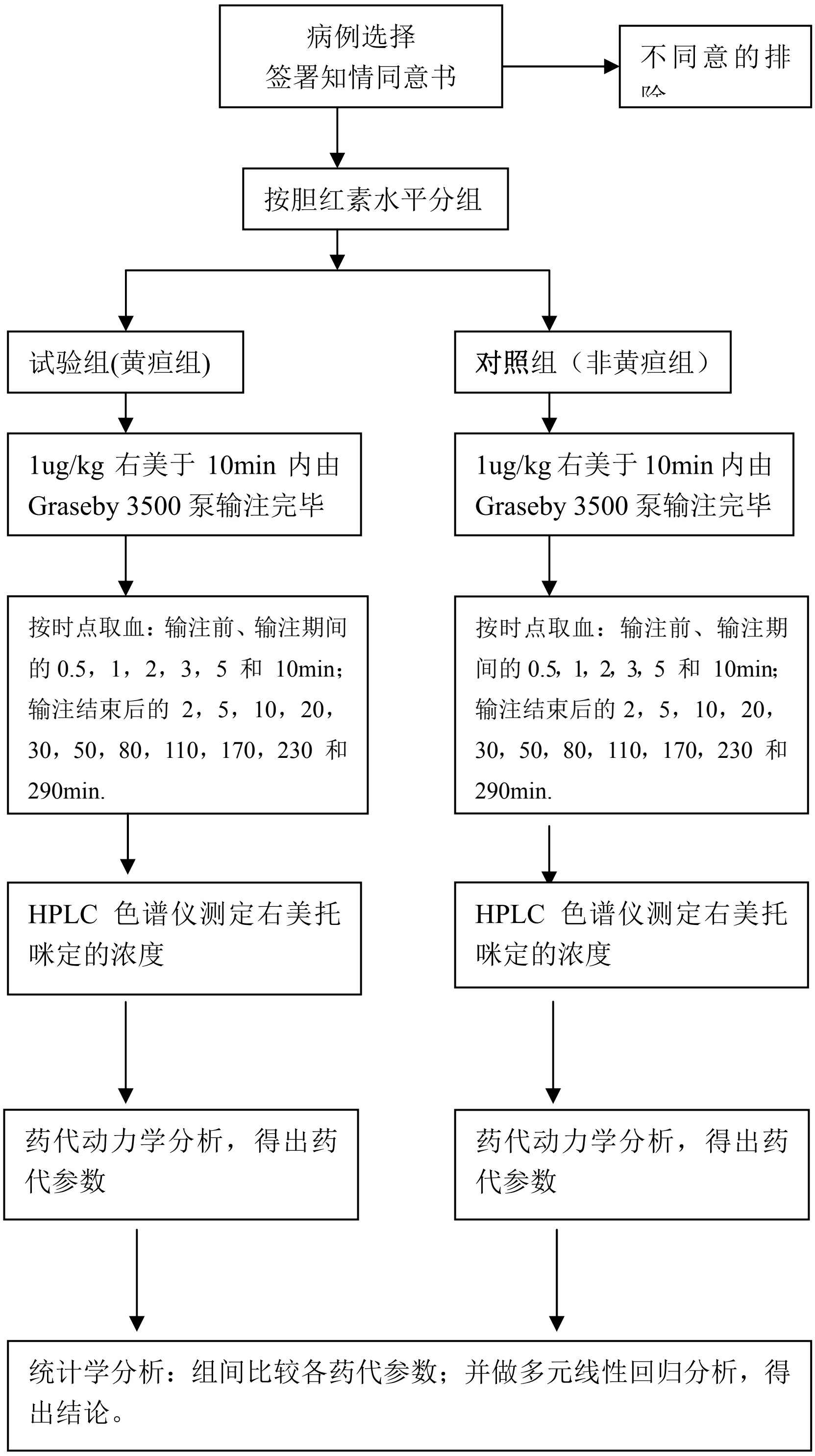

Supplement: S4 File — (PDF) [file pone.0207427.s004.pdf]
